# Redefining the polypill: pros and cons in cardiovascular precision medicine

**DOI:** 10.3389/fphar.2023.1268119

**Published:** 2023-09-19

**Authors:** Siddharth Birla, Arshia Angural, Arya Madathumchalil, Ritika V. Shende, Sharvani V. Shastry, Manjappa Mahadevappa, Sunil Kumar Shambhu, Prashant Vishwanath, Akila Prashant

**Affiliations:** ^1^ Department of Biochemistry, JSS Medical College, JSS Academy of Higher Education and Research, Sri Shivarathreeshwara Nagara, Mysuru, Karnataka, India; ^2^ Department of Medical Genetics, JSS Medical College, JSS Academy of Higher Education and Research, Sri Shivarathreeshwara Nagara, Mysuru, Karnataka, India; ^3^ Department of Cardiology, JSS Medical College and Hospital, JSS Academy of Higher Education and Research, Sri Shivarathreeshwara Nagara, Mysuru, Karnataka, India

**Keywords:** polypill, LDL, hypertension, CVD, NAFLD, multi-drug, precision medicine, genomics

## Abstract

Polypill is a multi-drug formulation in a single pill intended to simplify the drug regimen and reduce medication-induced adverse effects. The most common multidrug combinations in a polypill are used to treat cardiovascular diseases and are targeted against key modifiable risk factors such as hypertension and hyperlipidemia. These contain blood-pressure lowering agents, statins, and anti-platelet agents usually in a fixed dose. Polypills can be an affordable therapeutic intervention for treating high-risk patients, as these are proven to increase patients’ adherence to medication and improve clinical outcomes. Over the previous years, randomized clinical trials of several polypills have yielded contradictory findings, raising skepticism regarding their widespread use in primary disease prevention. Here, we have reviewed the concept of polypills, the evidence-based strengths, the limitations of this polypharmacy intervention strategy, and discussed future directions for their use in the primary and secondary preventive management of cardiovascular diseases and associated risk factors.

## 1 Introduction

Cardiovascular diseases (CVDs) are the leading cause of disability and premature death, contributing to the highest morbidity and mortality rates globally, especially in low- and middle-income countries (LMICs). Moreover, the CVD-associated morbidity rate has been projected to enhance, surpassing that of communicable diseases (Laslett et al., 2012). Despite the availability of different cardiovascular (CV) medicines, a significant gap exists in the therapeutic management of CVDs and the prevention of associated risk factors. The most critical challenge faced in the therapeutic management of CVD patients is their poor compliance with the recommended therapy and lifestyle changes. This is mainly attributed to patient’s confusion about the consumption of complex therapeutic regimens consisting of a large number of recommended medicines or their antipathy towards these pills, and the affordability of the prescribed medication (Castellano et al., 2014). Furthermore, only some patients are able to receive a full combination of the recommended medicines (Lawlor et al., 2003). Some of the CVD patients may develop recurrent CV event (CVE) within a few years of their first CV episode despite undergoing preventive pharmacotherapy. All of these factors have been linked to poor clinical outcomes in the patients (Perel et al., 2015). However, the occurrence of these can be reduced by the use of preventive fixed-dose combinations (FDCs) of CV drugs, called Polypill, and multiple target medicines which may not increase pill burden among patients. Polypill has been defined as the FDC of several drugs that can control modifiable CVD risk factors or associated disease pathologies (Fuster et al., 2017).

The concept of developing combination therapy formulations or cardiovascular polypill (CVP) as a preventive therapeutic approach was originally proposed in 2001 by the World Health Organization (WHO) and Welcome Trust Expert Group (World Health Organization, 2002). The expert members suggested once-a-day oral administration of a single combination pill containing four drugs including a β-blocker, an angiotensin-converting enzyme inhibitor (ACEI), a statin, and aspirin, each well-documented for their preventive roles in CVD. Later, Yusuf *et al.* reported that there could be a 75% reduction in CVEs upon implementation of a combination of four drugs (aspirin, beta-blocker, statin, and angiotensin-converting enzyme (ACE) inhibitor) in CVD patients (Yusuf, 2002). They had put forward a precept that the first heart attack or stroke event could be prevented by using cumulative effects of various drugs in combination as a single pill or polypill. An FDC of antihypertensive agents, aspirin, lipid-lowering drugs, and sometimes folic acid has been suggested for the wellbeing of patients with a high CVD risk. Nicholas Wald and Malcolm Law suggested six different drug components in a polypill, originally including aspirin, ACE inhibitors, beta-blockers, diuretics, folic acid, and statin. An analysis by Wald and Law demonstrated that there is a drop in myocardial infarction and stroke rates by over 80% when their model of polypill was assessed in individuals aged above 50 years (Wald and Law, 2003). This polypill model could improve platelet function and regulate blood pressure (BP), low-density lipoprotein cholesterol (LDL-C), and serum homocysteine levels within the optimum biological range in CVD-risk patients (Table 1). Purportedly, this formulation can reduce the risk of ischemic heart disease (IHD) and stroke by 46% and 63%, respectively, whereas statins can lower the mean LDL-C levels by 1.8 mmolL^-1^, decreasing IHD risk and stroke by 60% and 17%, respectively (Law et al., 2003b). The CVPs were, thereby, recommended by them to improve the key CVD risk factors.

**TABLE 1:** The table reveals the outcomes of Wald and Law’s cardiovascular polypills on ischemic heart disease and stroke after 2 years of treatment (Wiley and Fuster, 2014).

Risk factor	Drug agents	Reduction in risk factor	
		IHD (in %); 95% CI	Stroke (in %); 95% CI
Blood pressure	Three different classes of antihypertensive^#^ drugs at a half-standard dose	46; 39–53	63; 55–70
LDL-C	Statin^*^	61; 51–71	17; 9–25
Serum homocysteine	Folic acid (0.8 mg/day)	16; 11–20	24; 15–33
Thrombosis	Aspirin (75 mg/day)	32; 23–40	16; 7–25
Combined effect		88; 84–91	80; 71–87
Combined effect (omitting folic acid)		86	74
^#^Thiazides, beta-blockers, angiotensin-converting enzyme (ACE) inhibitors, angiotensin II receptor antagonists, and calcium channel blockers			
^*^Atorvastatin (10 mg), Simvastatin, or Lovostatin (80 mg taken in the morning or 40 mg taken in the evening)			
Abbreviations: LDL-C = Low Density Lipoprotein Cholesterol; IHD = Ischemic Heart Disease; CI = Confidence Interval			

A fixed half-dose formulation of multiple antihypertensive drugs is reported by several studies to lower the elevated BP and other associated risk factors with relatively less adverse effects in comparison to a full dose. Chief health organizations like the Centers for Disease Control (CDC), the WHO, and the Wellcome Trust have initiated research programs emphasizing the development of polypills and the evaluation of their effects (Combination Pharmacotherapy and Public Health Research Working Group, 2005; Lonn et al., 2010). The European Commission has recently concluded lack of patient adherence to medication as one of the key contributory factors in persistent CVEs, and strongly advocated the use of polypills to address this problem (Fuster et al., 2017). Subsequently, CVPs are being scrutinized as an alternative therapy for improving therapeutic outcomes for CVD patients. In 2018, the European Society of Cardiology provided clinical guidelines on making CVP as an integral part of the comprehensive secondary prevention of CVD (Ibanez et al., 2017). The 2018 European Society of Hypertension and the 2018 and 2020 updates of the American Heart Association Hypertension guidelines have further endorsed the use of CVPs in the CVD management (Whelton et al., 2018; Williams et al., 2018; Williams et al., 2020). The first CVP was developed by Centro Nacional de Investigaciones Cardiovasculares (CNIC)—Ferrer, Europe, which recently became accessible as a general prescription for ameliorating CVD (Fuster et al., 2017).

Being an alternative to the already existing complex multi-drug regimen, combining or scaling up a package of individual anti-CVD drugs into a polypill has several benefits. The cost-effectiveness and simpler usage of polypills improve patient compliance with the treatment since these are widely accepted by the patients (Gnanenthiran et al., 2023). As these do not require any dose adjustment, the FDC in polypill makes it safer to use for controlling BP (Muñoz et al., 2019).

## 2 The combination therapy: polypill

A polypill is usually defined as an FDC of several pharmaceutical components in variable compositions that have demonstrated benefits against CVD and the associated modifiable secondary complications without significant adverse effects (Wald and Law, 2003). Many studies have reported the use of polypills in both primary as well as secondary prevention of CVD risk factors. These usually contain aspirin (50–125 mg), a potent statin (10 mg atorvastatin or 40–80 mg simvastatin), folic acid (0.8 mg), and three blood pressure-lowering drugs (beta blockers, angiotensin receptor blockers, ACE inhibitors, calcium channel blockers, and thiazide diuretics) at half the standard dose (Wald and Law, 2003; Lonn et al., 2010). Furthermore, polypills can be either multi- or single-purpose based on their scope of action against CVD-associated risk factors. A single FDC can be either targeted against each major CVD risk factor (known as a multi-purpose polypill) or can control only one risk factor (known as a single-purpose polypill) (Sukonthasarn et al., 2021).

Polypills can prevent CVD in a population-wide setting, owing to their potential advantages over conventional therapies, as mentioned elsewhere (Law et al., 2003a). The usage of a polypill, such as a gelatin capsule securing losartan (25 mg), atorvastatin (10 mg), hydrochlorothiazide (12.5 mg), and amlodipine (2.5 mg), has been recently demonstrated to cause a reduction in LDL-C levels and systolic BP (Muñoz et al., 2019). In the past few years, several polypills with different compositions, at least containing one antihypertensive, lipid lowering drug with or without aspirin, have been developed evaluated in different clinical trials, registered and marketed for secondary prevention of CVDs (Patel et al., 2022). A list of some of these CVPs is provided in Table 2.

**TABLE 2:** A list of some cardiovascular polypills that have undergone clinical trials previously and the potential inter-compositional interactions.

Brand name	Manufacturer	Constituents	Potential compositional (inter-drug) Interactions^*^
Polycap™	Cadila Pharmaceuticals Ltd., India	aspirin (100 mg), atenolol (50 mg), hydrochlorothiazide (12.5 mg), ramipril (5 mg), simvastatin (20 mg)	Aspirin may decrease the antihypertensive activities of Atenolol
			Aspirin may decrease hydrochlorothiazide’s excretion rate, which could result in a higher serum level
			The metabolism of aspirin can be decreased when combined with Simvastatin
			The therapeutic efficacy of Atenolol can be increased when combined with hydrochlorothiazide
			Atenolol may decrease Simvastatin’s excretion rate, which could result in a higher serum level
			Ramipril may increase the hypotensive activities of Atenolol
Polypill	Cipla, India	amlodipine (2.5 mg), hydrochlorothiazide (12.5 mg), losartan (25 mg), simvastatin (40 mg)	The serum concentration of Simvastatin can be increased when combined with amlodipine
			Simvastatin may decrease losartan’s excretion rate, which could result in a higher serum level
PolyIran	Alborz Darou Pharmaceutical Company, Iran	aspirin (81 mg), enalapril (5 mg); or	The therapeutic efficacy of Enalapril can be decreased when combined with aspirin
		atorvastatin (20 mg), hydrochlorothiazide (12.5 mg), valsartan (40 mg)	The excretion of atorvastatin can be decreased when combined with Valsartan
Ramitorva^®^	Zydus Cadila, India	aspirin (75 mg), ramipril (5 mg), atorvastatin (10 mg)	no compositional inter-drug interactions
Red Heart Pill^™^ 1	Dr Reddy’s Laboratories, India	atenolol (50 mg), aspirin (75 mg), lisinopril (10 mg), simvastatin (40 mg)	Aspirin may decrease the antihypertensive activities of Atenolol
			The metabolism of aspirin can be decreased when combined with Simvastatin
			The therapeutic efficacy of Lisinopril can be decreased when combined with aspirin
			Atenolol may increase Lisinopril’s hypotensive activities and decrease Simvastatin’s excretion rate, which could result in a higher serum level
Red Heart Pill^™^ 2	Dr Reddy’s Laboratories, India	aspirin (75 mg), hydrochlorothiazide (12.5 mg), lisinopril (10 mg), simvastatin (40 mg)	Aspirin may decrease hydrochlorothiazide’s excretion rate, which could result in a higher serum level
			The therapeutic efficacy of Lisinopril can be decreased when combined with aspirin
			The metabolism of Aspirin acid can be decreased when combined with Simvastatin
Starpill^®^	Cipla, India	aspirin (75 mg), atenolol (50 mg), atorvastatin (10 mg), losartan potassium (50 mg)	Aspirin may decrease the antihypertensive activities of Atenolol
			The risk or severity of renal failure, hyperkalemia, and hypertension can be increased when losartan is combined with aspirin
			Atenolol may decrease the excretion rate of atorvastatin, resulting in a higher serum level
			Atenolol may increase the arrhythmogenic activities of losartan
			The metabolism of atorvastatin can be decreased when combined with losartan
Trinomia^®^/Sincronium^®^	Ferrer Internacional, Spain/Hexal, Germany	aspirin (100 mg), atorvastatin (20 mg), ramipril (2.5, 5 or 10 mg)	no compositional inter-drug interactions
Trinomia^®^	Ferrer Internacional, Spain	aspirin (100 mg), ramipril (2.5, 5 or 10 mg), simvastatin (40 mg)	The metabolism of aspirin can be decreased when combined with Simvastatin
Zycad™	Zydus Cadila, India	aspirin (75 mg), atorvastatin (10 mg), metoprolol (50 mg), ramipril (5 mg)	Aspirin may decrease the antihypertensive activities of Metoprolol
			Ramipril may increase the hypotensive activities of Metoprolol
*Information is retrieved from DrugBank Online tool (URL: https://go.drugbank.com/)			

## 3 Issues to be addressed before implementing the polypill

Before the therapeutic implementation of the polypill, there must be a substantiation that it would cut down the occurrence of major CV illnesses and be safe enough for use as primary prevention in middle-aged individuals. Ideally, the multicombination drug has four to five active drug components in the form of an FDC. Therefore, the pharmacokinetics, bioavailability, risk factors, and adverse drug reactions (ADRs) as well as potential inter-drug interactions need to be documented for individual compositions of the polypill formulation, even though the effects of the polypill’s components, when combined, are seen to be cumulative (Patel et al., 2010). However, using Polypill does not contradict the effect of individualized treatment, rather it is considered as an appended therapy for persistent elevation in BP or LDL-C levels (Lonn et al., 2010). Certain factors are vital to the successful implementation of any FDC, including the cost- and dose-effectiveness of drugs used to formulate the polypills and their safe implementation based on the physicochemical compatibility within the individual components of the pill. Other considerable factors include effortless treatment methods, dosing time, intake of pills, and their respective side effects (Smith, 2009). The potency and safety of these drugs can be determined through clinical trials focussed on understanding the cumulative effects of the FDCs. Most widely acknowledged national and international therapeutic guidelines are now focussing on BP lowering and LDL-C regulating therapeutic objectives as a result of confirmed positive results from several clinical trials on drugs lowering the BP- and LDL-C levels (Sukonthasarn et al., 2021).

The CVPs have practically remained underutilized in clinical settings across the globe. This has been attributed to different issues including the physician’s personal perspectives on CVPs due to lack of established evidence base, their practical inexperience in using combination therapies and inability to titrate individual drug in order to achieve desired therapeutic outcome, some patient factors and barriers to health system (Webster et al., 2020; Patel et al., 2022; Gnanenthiran et al., 2023; Khan et al., 2023).

## 4 The pros and cons of polypill

Since the first description of the polypill concept, several clinical trials with different CVPs have demonstrated improved medicine adherence among patients and therapeutic outcomes compared to conventional therapies. Polypills have been reported as an effective alternative and preventive (primary and secondary) therapeutic measure by many researchers.

Initially, the international Heart Outcome Prevention Evaluation-3 (HOPE-3) study conducted in three phases evaluated the role of three drugs in over 1,200 participants with at least one CV risk factor, including men over 55 years and women over 65 years. The study did not report any significant reduction in the primary outcome of CV mortality, non-fatal myocardial infarction or stroke in patients receiving only BP-lowering therapy (a low dose of a candesartan/thiazide diuretic hydrochlorothiazide) compared to those receiving placebo (Lonn et al., 2016). Whereas, the participants who received only the statin (rosuvastatin 10 mg) showed a relative lower risk of CVEs by 24% compared to those receiving placebo (Yusuf et al., 2016a). The participants who received the combination of two hypertensive drugs and the statin displayed significantly lower rates of the primary outcome compared with those receiving double placebo (Yusuf et al., 2016b).

The PolyIran study had previously indicated that the use of polypill significantly helps in the primary prevention of CVD, reducing the risk of major CV events in individuals with or without any previous CVD history (primary prevention) by 20% and 40%, respectively (Roshandel et al., 2019). Although this analysis did not show any significant interaction, the results might suggest that the use of polypills could be considered for primary prevention. Findings of a randomized clinical trial (RCT), named The International Polycap Study 3 (TIPS-3), revealed that participants with intermediate CV risks but without CVD, who received combined treatment with polypill and aspirin had better CV outcomes than placebo (Yusuf et al., 2020). A recent international, multi-centric, randomized phase-III clinical trial of the CNIC-Polypill, named the VULCANO study, conducted for 16 weeks in 499 subjects (at high or very high risk without a previous CVE) from 47 centres assessed the impact of this multi-drug formulation on LDL-C and systolic BP levels (Mostaza et al., 2022). This study reported the CNIC-Polypill as “safe-to-use” and can be a suitable multi-drug therapeutic strategy in preventing CVD and controlling associated risk factors.

Furthermore, the findings of the FOCUS (Fixed Dose Combination Drug for Secondary Cardiovascular Prevention) project have highlighted the role of FDC in the secondary prevention of CV events (Castellano et al., 2014). A recent observational retrospective study, the NEPTUNO study, assessed the effectiveness of the CNIC-Polypill in secondary prevention. The study findings suggested a significant reduction in the incidence of recurrent major adverse CVEs (MACE), total cholesterol and systolic BP in a group of patients with established atherosclerotic CVD and who were on the polypill treatment (test group) when compared with those on monotherapy (González-Juanatey et al., 2022). Other therapeutics outcomes such as improved blood pressure and medicine adherence in the test group were better compared to the study control groups. In a yet another RCT study named the SECURE (Secondary Prevention of Cardiovascular Disease in the Elderly) trial, efficacy of a polypill containing aspirin (100 mg), ramipril (2.5, 5, or 10 mg) and atorvastatin (20 or 40 mg) was assessed with respect to medication adherence and major CV outcomes in 2,499 patients aged over 65 years who had Type I myocardial infarction (Castellano et al., 2022). The authors suggested a significant risk reduction in MACE among those recruited subjects, who had a relatively higher rate of adherence to the trialled polypill.

Recently, a number of meta-analyses on these RCTs suggested the overall effectiveness or outcomes of polypill therapy in medicine persistence, prevention and therapeutics of CVDs. One such study by Rao et al. (2022) evaluated polypill’s influence on the CVEs and associated risk factors (Rao et al., 2022). This study supported the fact that the usage of CVPs significantly improve adherence to medication without any association between their use and rates of adverse events or drug discontinuation. It was further reported that their use results in risk reduction for MACE among low-risk and primary prevention patients, overall reduction in CVD risk factors and the risk of all-cause mortality. Another meta-analysis of RCTs by Mohamed et al. (2022) reported a positive association between the use of polypills and reduction in levels of clinical risk factors including BP (both diastolic and systolic), total cholesterol, LDL-C, CVD mortality and MACEs (Mohamed et al., 2022). Furthermore, Hennawi et al.’s (2023) meta-analysis of 18 RCTs with a total participation of 20,463 individuals evaluated the efficacy of polypill therapeutic approach in reducing CV risk factors such as hypertension and dyslipidemia (Hennawi et al., 2023). This study reported a statistically significant association between polypill therapy and improved medicine adherence as well as reduction in BP (both systolic and diastolic) and total cholesterol levels in high-risk individuals or those with confirmed CVD diagnosis. Initially, the concept of using a polypill in CVD management led to conjectures (Smith, 2009).

Although there are several benefits to the intake of polypills, it has also been proven detrimental in many aspects (Webster et al., 2020). A few studies have also pointed to relative contraindications to polypills. Recently published findings of the TIPS-3 clinical trial failed to provide any significant evidence of the polypill’s positive impact on improving cognitive and functional decline in people aged over 65 with CV risk factors (Bosch et al., 2023). This polypill, with or without aspirin, was associated with reduced functional decline in these patients without causing any detrimental effects on cognition. The MACEs, such as cardiac death, myocardial infarction, stroke, acute coronary syndrome, revascularisation procedures, development or worsening of heart failure, and development of persistent new arterial fibrillation estimated for 34 months, are some of the other primary outcomes of polypill’s randomized clinical trials (Sadeghi et al., 2022). Furthermore, previous clinical trials on CV polypills were conducted on populations from LMICs and the underprivileged population in high-income countries (Muñoz et al., 2019). Though these clinical trials had provided encouraging results in enhancing patient-medication adherence rates, alleviation of CVD risk factors, and fewer adverse events, most of these studies (except the PolyIran Trial) lacked enough statistical power for evaluating the impact of polypill on the clinical outcomes of CVD patients.

Having two sides to the same coin, polypills are not only proven beneficial but there are certain limitations to their use. These include difficulties with dose adjustment to focus on target patients, concern about the consumption of unnecessary medication, and management of too low BP or LDL-C levels. Titration adjustment of individual components within the polypill can be another major challenge while adjusting dose for co-morbid patients, elderly patients, and patients already taking multiple medications. In addition to contradictory evidence for the role of CVPs in improving the clinical outcomes of CVD patients, there are further concerns about their unknown potential adverse consequences (such as bleeding events, and dizziness) in clinically healthy populations taking these pills as a preventive measure. The aforementioned factors can impact the clinical application of polypills for managing BP and other conditions, as these may preclude the recommendation of CVPs by clinicians, subsequently making the patients opt for risk-based therapies with lower treatment thresholds.

Further, there are several additional challenges to the application of polypill, some of which include the technical issues faced while developing an FDC, regulatory hurdles for poorly defined combinations of three or more components, lack of research and development budgets on FDC formulations, and their clinical trials, the unwillingness of a few companies to invest because of the low-profit gains, the ambiguities connected with intellectual property, and other regulatory and fabrication issues (Rafter and Woodward, 2005). Moreover, Walde and Law’s statement about taking the polypill by those over 55 years is way too generalized and remained controversial (Wald and Law, 2003). It is not justified whether the polypill is intended for general public use or only for specific populations with a relatively high burden of CVD. It is further questionable whether the individual benefits of each drug component of the polypill are cumulative in the affected populations, and whether a one-sized FDC is effective and safe for everyone without any adverse effects. Although the individual adverse reactions of each component in a polypill are well known (as highlighted in Table 2), it is a challenge to decipher which drug component might have caused an adverse reaction in a suspected drug-related adverse event. Previously conducted clinical trials on one or other polypills assessing the role of adding an extra class of drug component have revealed that these do confer a risk reduction. But none of these trials were based on studying the exact composition like the polypill proposed by Walde and Law. Despite all these, a genre of mini polypills is available (Rafter and Woodward, 2005).

## 5 Polypill usage in hypertension and NAFLD patients

It has been noted that patients with hypertension and non-alcoholic fatty liver disease (NAFLD) are more vulnerable to major CVDs. Hypertension is a major global health issue and a leading preventable risk factor for premature death and disability worldwide. The swift switch from monotherapy to combination medication is part of the strategy to improve the management of BP. For achieving comparable management of hypertension, multi-drug treatments in a low therapeutic dose have been documented to be more well-tolerated than the corresponding monotherapies in a higher dose (Law et al., 2003a; Wald et al., 2009; Mancia et al., 2014). Furthermore, several recent clinical guidelines have advised multimodal therapy with a combination of two BP-lowering drugs taken in the form of a single pill as the first therapy for the majority of hypertensive patients (Gupta et al., 2010; Sherrill et al., 2011; Mancia et al., 2014; Weber et al., 2014; Lafeber et al., 2016; Brainin et al., 2022). At a broader outset, the polypill approach has been shown to prolong treatment adherence relative to usual care in all CVD patients, while also suggesting a further reduction in concomitant risk factors (Cimmaruta et al., 2018).

NAFLD is the most typical chronic liver ailment prevalent in developed countries. It is assessed that NAFLD will be the leading and one of the foremost causes of liver transplantation by the year 2030 (Tana et al., 2019). Except for the recognized liver-associated morbidity and mortality, a large body of evidence shows that CVD risk represents the foremost cause of death in NAFLD patients (Tana et al., 2019). Recently a 5-year extended clinical trial, named PolyIran-Liver Trial, conducted in a randomized population setting in Iran, investigated the effectiveness of a polypill (aspirin, atorvastatin, hydrochlorothiazide, and valsartan) for the deterrence of MACEs among individuals with and without presumed non-alcoholic steatohepatitis (pNASH). The primary findings of this trial suggested that NAFLD patients consenting to receive the FDC medication had a better outcome against MACE, indicating polypill to be safe and effective against fatty liver disease and enhanced liver enzyme levels (Merat et al., 2022). The use of this multi-drug formulation indeed benefitted NAFLD subjects without pNASH by alleviating the levels of liver enzymes, whereas no changes were observed for those with pNASH after a 60-month follow-up. On the contrary, its administration in participants either with or without pNASH could not indicate any statistical significance in lowering the risk of MACE. The results further indicated that polypill had significantly reduced Alanine Transaminase (ALT) levels in individuals with NAFLD-pNASH.

## 6 FDC in the welfare of underprivileged, vulnerable groups

A growing number of CVD incidences in LMICs calls for simple and cost-effective therapeutic approaches for primary as well as secondary prevention of CVDs. A polypill-based therapeutic strategy can aid all the communities, especially the most vulnerable and underprivileged ones with fewer resources and facing a relatively higher burden of CVDs and CVD-related mortality. Therefore, these communities must be given primary access to polypills.

It has been proposed by Lim et al. (2007) that almost a fifth of all deaths from CVD could be averted by scaling up a prevention approach based on opportunistic screening, identification of a high-risk individual, treatment with a multi-drug regimen, and following a moderate increment in health expenditure (Lim et al., 2007). A simple FDC can help tackle CV health disparities by possibly improving the patients’ adherence to medication and reducing the need for dose adjustments and multiple clinic visits (Muñoz and Wang, 2019). The CVPs can be a cost-effective, feasible, and overall attractive option in the improvement of CV health than targeting individual risk factors (Yusuf, 2002; Lim et al., 2007). In the long term, these can help in reducing the healthcare costs (Roebuck et al., 2011; Becerra et al., 2015; Coca et al., 2023). Based on a wide range of factors, the cost of polypill has been anticipated between $0.06-$0.94/day (Singh et al., 2018). Studies have further provided evidences on cost-effectiveness of the recently trialled CNIC-Polypill (Becerra et al., 2015; Cordero et al., 2021). The economic analyses in the recent NEPTUNO study carried out on 6,456 patients in Spain has shown that the CNIC-Polypill used for secondary CV prevention is cost-effective compared to monotherapy and its use incurs a relatively lower utilization of the healthcare resources and total costs (González-Juanatey et al., 2022; Cordero et al., 2023). This further provided information on the associated cost-savings ranging between €17,790 to €26,257 per patient without CVE. This model reflected on significant increments in the patients’ living years and quality of life. Furthermore, an economic model developed under the Portuguese MERCURY study evaluated the cost-effectiveness and public health benefits of the CNIC-Polypill compared to other alternative therapies (Aguiar et al., 2022).

Implementation of the Polypill therapeutic approach can be challenging, especially in the LMICs, given the constraints of low income, multiple clinical visits, testing and dose adjustments, and under-insurance of individuals in these countries. But these challenges are somehow manageable. The governments and policymakers in individual LMICs and other countries must ensure relatively easy access and ample supply of CVPs at nominal prices to the eligible individuals in their countries. On the other hand, the use of polypills can further be limited by eligibility determinants regarding polypill users. They should be above 55 years and have a history of CVD, systolic blood pressure ranging between 120 and 160 mmHg, LDL cholesterol <190 mg/dL, a glomerular filtration rate of at least 60 mL/min, less hepatic amino-transferase levels, normal potassium level, no contradictions to the polypill components, and using no more than two antihypertensive medications (Muñoz et al., 2019; Castioni et al., 2021). However, the use of CVPs in children and pregnant women as a preventive therapy is not recommended as some of the drug compositions (such as ACE inhibitors, anti-hypertensive drugs, statins) are contraindicated for their use (Alexander et al., 2009; JCS Joint Working Group, 2014; Kaelber et al., 2016; Mehta et al., 2020).

## 7 Tailoring the dose of individual polypill components

Recently in clinical practice, there has been a rising interest in precision medicine, particularly in pharmacogenetics and implementation of the genomic medicine. Studies have demonstrated a significant inter-individual variability in terms of drug response, which has been mainly associated with their specific genetic profiles or genotypes of CVD-related Pharmacogenes, influencing drug metabolism, drug transport, and drug effects (Vrablik et al., 2021). Lately, technological advancements have ushered the field of Pharmacogenetics and Pharmacogenomics and enabled the scientific community to correlate genetic variations with the efficacy and/or toxicity of anti-CVD drugs, facilitating the identification of individuals who could have a better response or are poor responders at a greater risk of developing adverse responses to a particular pharmacotherapy. Identification of such pharmacogenetic variants would further allow the consumption of relevant drugs at optimal dosing, thereby, improving the health outcomes of CVD patients. Although seems challenging, tailored dosing of individual drug components according to the patient’s genetic profile would be intriguing and effective in enhancing the therapeutic outcomes of polypills. For further reference, information on some of the genetic variants associated with individual drug compositions of clinically trialled polypills and their responses is provided in Supplementary Table S1. Though in the context of personalized medicine, it is still questionable whether fine-tuning the polypill components should be population-specific or personalized. However, this would require further shreds of evidence.

## 8 Future directions

The use of CVPs in the preventive management of CVDs is on the rise lately. These are considered safe and highly effective secondary preventive medications for reducing the incidence of major CVDs. Studies have shown that three-quarters of the incidence of heart attacks and strokes could be averted if these are taken regularly for a longer period. Since the polypills have a simple regimen as a single-pill FDC, these have been proven beneficial in improving patients’ adherence to medication with better clinical outcomes. CVPs have extensive potential of reducing the burden of CVDs, especially in LMICs, because of their cost-effectiveness and lesser side effects. The CVPs could correspond to a strategic therapeutic solution in hypertension, co-morbid, and non-adherent patients, even though further studies are required to understand their role in clinical practice. Though limited in number, recent clinical trials evaluating the efficiency of these CVPs have proven instrumental in providing information on their effectiveness in disease management and patient compliance to medication as well as their safety. Yet further studies are required to assess the impact of polypill-based therapy on hard clinical outcomes of CVD such as mortality and MACEs. Although CVPs are being considered for inclusion into the WHO List of Essential Medicines, there is still a long way to fully assess their potential in alleviating CVD-associated risk factors. Scaling up the polypill-based intervention is essential for reducing CVD-related mortality in the coming years. Demonstrating the potential health effects of CVPs and the cost of scaling up the required resources would influence relevant agencies to set an appropriate action plan to mobilize essential resources by developing necessary financial as well as infrastructural aids. At the most, the success of the recent clinical trials should motivate a large-scale implementation of polypills with all possible outcomes. To have a consensus on tailored dosing of individual polypill components, nevertheless, further evidence from more population-specific clinical trials targeting a larger population based on the genotype of relevant Pharmacogenes is a need-of-the hour. Maintaining a positive mindset in the public interest that does not impede the availability of cost-effective health interventions due to personal profit issues would greatly improve the outcomes of CVP-based intervention worldwide, especially for the LMIC populations. As Lonn et al. (2010) proposed, an individual high-risk intervention strategy should complement a population-based intervention approach to reduce risk factors in an entire population (Lonn et al., 2010). Ensuring patients’ access to affordable preventive medications like CVPs at a global level will substantially benefit them by improving their clinical outcomes.

In conclusion, polypill has a colossal clinical outcome in the primary and secondary management of CVD patients compared to monotherapies, despite a few complications associated with potential side effects of their individual medicinal compositions. These have proven to enhance adherence rates and lower healthcare costs, but more research is still needed to determine their safety and efficacy. Further studies are warranted to determine whether polypills can be custom-tailored for personalized therapeutics.

## Abbreviations

ADRs, Adverse Drug Reactions; ACE, Angiotensin-Converting Enzyme; ALT, Alanine Transaminase; BP, Blood Pressure; CDC, Centers for Disease Control; CV, Cardiovascular; CVD, Cardiovascular Disease; CVE, Cardiovascular Event; CVP, Cardiovascular Polypill; CNIC, Centro Nacional de Investigaciones Cardiovasculares; FDC, Fixed-Dose Combination; FOCUS, Fixed Dose Combination Drug for Secondary Cardiovascular Prevention; HOPE-3, Heart Outcome Prevention Evaluation-3; IHD, Ischemic Heart Disease; LDL-C, Low-Density Lipoprotein Cholesterol; LMICs, Low and Middle Income Countries; MACE, Major Adverse Cardiovascular Events; NAFLD, Non-alcoholic Fatty Liver Disease; pNASH, Presumed Non-Alcoholic Steatohepatitis; RCT, Randomized Clinical Trial; TIPS-3, The International Polycap Study 3; WHO, World Health Organization.
